# A rare occurrence of a malignant ovarian steroid cell tumor not otherwise specified: A case report and literature review

**DOI:** 10.3892/ol.2014.2233

**Published:** 2014-06-06

**Authors:** KAI LI, FUFAN ZHU, JING XIONG, FENGYING LIU

**Affiliations:** Department of Obstetrics and Gynecology, The Second Xiangya Hospital, Central South University, Changsha, Hunan 410011, P.R. China

**Keywords:** ovarian steroid cell tumor, not otherwise specified, bone metastasis, chemotherapy

## Abstract

Steroid cell tumors not otherwise specified (NOS) are a rare subgroup of sex cord-stromal tumors. The tumors can occur at any age, although the mean age of occurrence is 43 years old. The majority are benign, but have the capability of producing one or more steroids associated with virilization. The present study reports the case of a 29-year-old female who presented to the Second Xiangya Hospital suffering from lower back and leg pain that had persisted for five months. The patient had regular menstrual cycles and no virilization symptoms were present. Laboratory investigations revealed normal hormone levels. Multiple areas of bone destruction and a right ovarian mass were confirmed via positron emission tomography/computed tomography. The patient underwent an exploratory laparotomy, and a mass measuring ~6 cm in diameter was subsequently identified in the right ovary. A right salpingo-oophorectomy and pelvic washings for cytology were performed. Histopathological studies confirmed the diagnosis of a malignant steroid cell tumor NOS of the right ovary. The patient underwent eight cycles of chemotherapy (docetaxel, 120 mg and nedaplatin, 80 mg). The patient continued to have relatively good health, with no deterioration of the condition for one year and a half, however, the disease progressed and the patient succumbed to brain metastases six months later.

## Introduction

A description of ovarian steroid cell tumors was first provided in 1979 by Scully (1). The tumors belong to the sex cord-stromal tumors and account for <0.1% of all ovarian neoplasms (2). According to their cell of origin, these tumors have been divided into three subtypes: Stromal luteomas, Leydig cell tumors and steroid cell tumors not otherwise specified (NOS). The last group accounts for 60% of these tumors (3), which often produce steroids, particularly testosterone, and may present with virilizing symptoms, including hirsutism, a deep voice, baldness and amenorrhea. In total, ~25% of patients lack endocrine symptoms (4). Malignant tumors are extremely rare, with only ~31 reported cases found via a PubMed search of the English literature (http://www.ncbi.nlm.nih.gov/pubmed) (5–17). The present study reports a case of a malignant steroid cell tumor NOS in a patient younger than the mean age of occurrence, with lower back and leg pain as the initial symptoms, and with a lack of endocrine symptoms. Due to the lack of specific symptoms, a correct diagnosis was not formed pre-operatively, but by post-operative pathological examination. Various aspects of the presentation, diagnosis and treatment of this type of tumor are also discussed. Patient provided written informed consent.

## Case report

**Figure 1:**
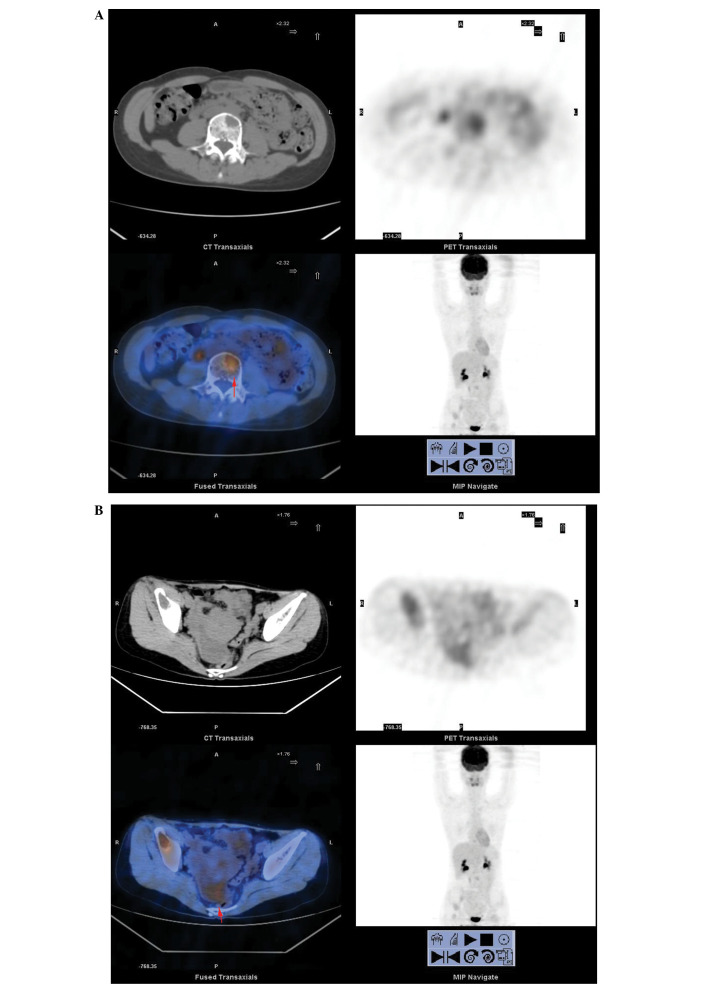
(A) Multiple bone destruction: Cystic destruction of the vertebral body. (B) A right-sided ovarian mass.

**Figure 2:**
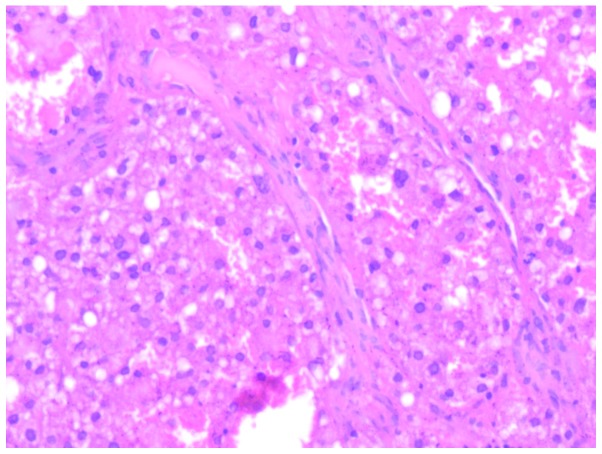
Eosinophilic cell type (stain, hematoxylin and eosin; magnification, ×20) with abundant eosinophilic granular cytoplasm and small to intermediate nuclei, with small nucleoli and distinct cell borders.

**Figure 3:**
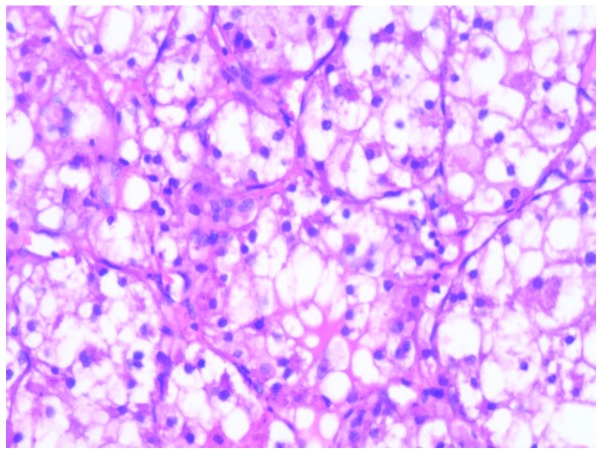
Clear cell type (stain, hematoxylin and eosin; magnification, ×20), rich in transparent cytoplasm, with visible large vacuole formation and small nuclei.

**Figure 4:**
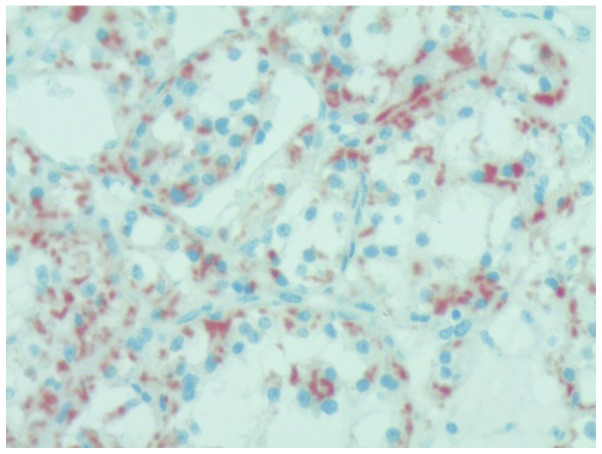
Immunohistochemical staining revealing chromogranin A (CgA) positivity in the tumor cells. Magnification, ×20.

**Figure 5:**
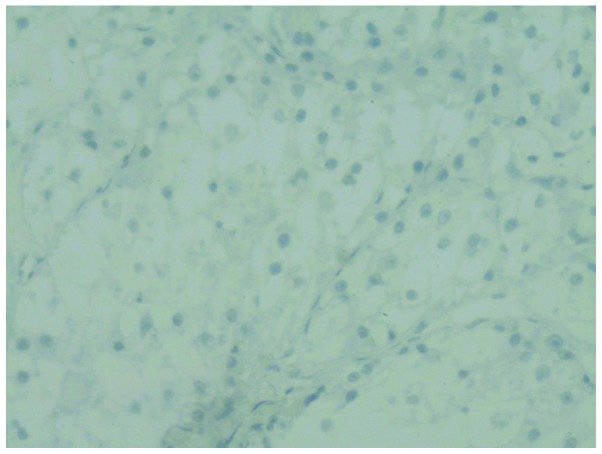
Immunohistochemical staining revealing a negative result for α-fetoprotein (AFP) staining in the tumor cells. Magnification, ×20.

A 29-year-old female presented to the Second Xiangya Hospital with complaints of lower back and leg pain that had persisted for five months, and a history of a ovarian mass that had been diagnosed upon presentation to the Hunan Provincial Tumor Hospital with the same complaints two months previously. The patient was admitted to the Second Xiangya Hospital for further management of this condition. The patient history revealed that menarche had occurred at 14 years old, and that regular menses lasting 5 to 6 days had occurred every thirty days since then. The family history was negative for any type of cancer. A pelvic examination revealed a 5×5×6-cm solid right adnexal mass. Laboratory workups revealed that the levels of sex and thyroid hormones, and 12 types of serum tumor markers, including cancer antigen (CA)-125 and CA19–9, were within the normal limits. Gastroscopy and colonoscopy findings were also normal. A transvaginal pelvic ultrasound showed a 57×36-mm solid-cystic mass of the right ovary. No ascites or other abnormalities were present. Whole body positron emission tomography (WBPET) and computed tomography scans (performed at the Hunan Provincial Tumor Hospital, Changsha, Hunan, China) found multiple areas of cystic destruction of the bones, which were highly suspected to be caused by malignant tumors (Fig. 1A). Previously, Glaspy *et al* (18) reported that PET imaging has the potential to demonstrate the biochemical differences between normal and malignant tissues to reveal primary or metastatic malignancy. A 33×64-mm solid-cystic mass was detected on the right in the rear of the pelvis, indicating the possibility of ovarian cancer (Fig. 1B). An ovarian tumor was suspected. The patient underwent an exploratory laparotomy; a small amount of yellowish ascites was observed intraperitoneally, but the exploration of the liver, spleen, kidney and greater omentum was normal. No lymphonodus was identified. The bilateral fallopian tubes, left ovary and uterus were normal. A well-encapsulated and solid 6×6-cm mass was located occupying 90% of the right ovarian tissue, without any adhesions to the surrounding structures. A right salpingo-oophorectomy and pelvic washings for cytology were performed. A frozen section of the mass revealed a malignant tumor, which could not be classified, with large areas of necrotic tissue. Nitrogen mustard (2%) was used to wash the abdominal cavity. The specimen was sent for histopathological examination. Macroscopically, the mass measured 60×58×57 mm in size and had an intact and smooth external surface. The cut surface was solid and yellow in color with multiple areas of necrosis. Upon microscopic examination, the tumor cells were diffusely arranged, with the tumor areas exhibiting two types of cells: i) Eosinophilic cells with abundant eosinophilic granular cytoplasm, small to intermediate nuclei with small nucleoli and distinct cell borders (Fig. 2); and ii) clear cells rich in transparent cytoplasm, visible large vacuole formation and small nuclei (Fig. 3). Regional cells were arranged in cords or nests. Hemorrhagic necrosis, nuclear atypical and mitotic figures were also apparent. Peritoneal fluid cytology did not reveal any malignant cells. Immunohistochemical staining revealed that neuron-specific enolase, chromogranin A (CgA; Fig. 4), cluster of differentiation (CD)68, CD34, placental alkaline phosphatase, inhibin A and S100 were positive, while smooth muscle actin (SMA), CD30, vimentin, α-fetoprotein (AFP; Fig. 5), epithelial membrane antigen (EMA) and cytokeratin were negative. The clinical findings, histomorphology and immunohistochemistry of the mass revealed a diagnosis of malignant ovarian steroid cell tumor NOS. The patient was administered chemotherapy (docetaxel 120 mg and nedaplatin 80 mg) on post-operative day 6. The patient received 8 cycles of the same chemotherapy regimen. The patient lived a relatively healthy life and there was no deterioration of the condition for a year and a half. However, following this, the disease progressed and the patient succumbed 6 months later from brain metastases.

## Discussion

It has been reported that ovary steroid cell tumors are rare and account for 0.1% of ovarian tumors (2). Steroid cell tumor NOS is a subtype of steroid cell tumors; their cell lineage is not defined and they cannot be categorized as either stromal luteomas or Leydig cell tumors. The tumors occur at any age, but with an mean age of 43 years (4), which is younger than that for other steroid cell tumors. Usually unilateral, only 6% of cases are found to be bilateral. The majority are benign, with 25–40% of patients with malignant tumors. The clinical manifestations of the tumor are associated with its hormonal activity and virilizing properties. Among the patients affected with the tumors, 56–77% have virilizing symptoms, including hirsutism, acne, clitoral enlargement, a deep voice and alopecia, and 6–23% have estrogenic manifestations, such as menorrhagia, postmenopausal bleeding or even endometrial cancer. Cushing’s syndrome occurs in only 6–10% of the cases, while gynecological examinations or surgery reveal that ~25% are not associated with hormonal disturbances (2,11,19–23). The patient in the present study was 29 years old, younger than the average age of occurrence, with an apparent absence of androgenic manifestations. Lower back and leg pain were the first symptoms, and a gynecological examination, transvaginal pelvic ultrasound and WBPET/CT located a right adnexal mass. Clinically, NOS tumors can present as abdominal pain, abdominal distention and bloating, however, to the best of our knowledge, metastatic bone pain as the first symptom has not previously been reported.

Pathological examination is an important method to diagnose steroid cell tumors. The gross specimen shows a tumor with a clear boundary, almost always well circumscribed and solid, with an enveloped, lobulated or nodular appearance (24,25). A total of 94% of cases are unilateral, while 6% are bilateral (2). The average diameter of the tumor is 8.5 cm, and the cut surface is yellow or orange (25), with occasional bleeding or cystic degeneration. Microscopically, the tumor cells are round or polygonal, and medium-to-large in size with distinct cell borders. The tumor cells often have central nuclei and prominent nucleoli. The neoplastic cells are usually of two types; eosinophilic and clear cells. The two types of cells differ only in their cytoplasmic appearance. The eosinophilic cells contain eosinophilic, slightly granular cytoplasm, while the clear cells have abundant vacuolated clear cytoplasm which is often positive for fat stains (2,16,20,25–27). In contrast to Leydig cells, the clear cells lack crystals of Reinke in the cytoplasm (26,27). The tumor cells are arranged predominantly in a diffuse pattern, but may be arranged in nests, clusters, columns or cords, separated by rich vascular characteristics. In the present case, the gross and microscopic findings were consistent with the characteristics of the ovarian steroid cell tumor NOS. The malignant cells show marked atypia, mitoses, necrosis and hemorrhage. The literature indicates that 25–43% of steroid cell tumor cases are clinically malignant, with 20% of cases found to exhibit metastases outside of the ovary during surgery (2,7). If metastasis is present, ~20% of metastatic lesions usually occur within the peritoneal cavity and rarely occur at distant sites. Although malignancy is often associated with metastasis, a study by Hayes and Scully (2) identified five pathological features predictive of malignancy: i) Two or more mitoses per 10 high-power fields (92% malignant); ii) a tumor diameter of >7 cm (78% malignant); iii) necrosis (86% malignant); iv) hemorrhage (77% malignant); and v) grade 2 or 3 nuclear atypia (64% malignant) (1). However, tumors with a benign histomorphology may be clinically malignant (17). The tumor of the current case was ~6 cm in diameter and hemorrhagic necrosis, nuclear atypia and mitotic figures were apparent. WBPET and CT scans found multiple areas of cystic destruction of the bones, which was highly suspected to be the result of malignant tumors. Due to these characteristics, the diagnosis of malignant steroid cell tumor NOS was determined. The most unusual feature in the present case was the bone metastasis, as it occurs infrequently in steroid cell tumors. Multiple bone metastases, without pelvic and lymph node metastasis, has not been previously reported. Another unusual feature is the apparent absence of endocrine manifestations; the patient’s hormone levels were normal. Other than the aforementioned microscopic features, immunohistochemical analysis aids the formation of a correct diagnosis. A number of immunohistochemical markers can be applied. The majority of steroid cell tumors are positive for calretinin and inhibin (28), while 75% of cases are vimentin-positive. The presence of HMB45 is inconsistent, however, other markers, such as EMA, cytokeratin, CD99 and S-100, have been reported to be positive, while CgA, LeuM1, AFP, carcinoembryonic antigen and periodic acid-Schiff have been reported to be negative (29). Positivity for CgA has never previously been reported in the English literature (4), However, CgA was found to be positive in the present case. This is a novel finding. In this case, the gross and microscopic findings were consistent with an ovarian steroid cell tumor NOS. Positivity for inhibin and negativity for SMA, AFP and EMA supported this diagnosis. The multiple metastases in the bone, the large areas of necrosis and the nuclear mitoses were indicative of a malignant nature.

The differential diagnosis of steroid cell tumor NOS includes stromal luteomas, Leydig cell tumors, luteinized thecomas, luteinized granulosa cell tumors, pregnancy luteomas and carcinomas, primary clear cell carcinomas, metastatic renal cell carcinomas and adrenocortical carcinomas. All these were excluded prior to reaching the diagnosis of a steroid cell tumor NOS.

The treatment of ovarian steroid cell tumors is primarily surgical. In a young patient, unilateral salpingo-oophorectomy is adequate for the treatment of a stage Ia tumor. Older females who do not want to preserve their fertility should undergo a total abdominal hysterectomy, bilateral salpingo-oophorectomy and complete surgical staging. As pathologically benign steroid cell tumors can behave in a clinically malignant manner, those patients who have high hormone levels prior to surgery require measurements of the sex hormone levels throughout. Patients with malignant tumors should immediately undergo debulking surgery, with post-operative chemotherapy or radiotherapy. In the present case, considering the patient had bone metastasis and that the tumor was confined to the ovary, with no pelvic metastases, a right salpingo-oophorectomy was performed. Due to the rarity of this type of tumor, little focus has been assigned to its study, and as the majority of these tumors are diagnosed in an early stage and do not recur or metastasize, the therapeutic value of chemotherapy and radiotherapy is poorly understood (30). Adjuvant chemotherapy regimens currently recommended for treatment are as follows: Bleomycin, etoposide and cisplatin; cisplatin, doxorubicin and cyclophosphamide; taxane and platinum; and bleomycin, vinblastine and cisplatin (31–34). The most effective regimen has not yet been found, with the majority reporting poor efficacy. In the present study, with treatment using a docetaxel and nedaplatin regimen, the patient survived for two years with multiple bone metastases. In recent years, the application of gonadotropin releasing hormone analogue has been attempted in order to treat this disease (35); gonadotropin releasing hormone agonist can restrain hormone secretion and induce cell apoptosis. This therapy has been attempted in patients who cannot tolerate surgery, or in the case of recurrence. This treatment method is only considered as an experimental therapy. However, it could provide a feasible treatment method and is worth further research.

In conclusion, ovarian steroid cell tumors NOS are rare, accounting for 60% of ovarian steroid cell tumors. The majority are benign, unilateral and usually characterized by virilizing symptoms. Pathological evaluation is essential for the diagnosis of malignancy, and immunohistochemical testing also aids in the formation of an accurate diagnosis. Surgery is the main treatment method and little is known about the response of the tumors to therapies such as chemotherapy or radiation. The present case is unique, as the patient was young, with an apparent absence of endocrine manifestations. The pain caused by bone metastasis as the first symptom has not been reported previously. Another unusual finding in this case is that the tumor was confined to the ovary, with no pelvic and lymph node metastasis, but with multiple bone metastases found by PET/CT. Additionally, CgA staining was found to be positive in this case, which was not consistent with the reported literature. The patient underwent a right salpingo-oophorectomy and chemotherapy of carboplatin and paclitaxel regimen for 8 cycles, and subsequently survived for two years. The patient lived a relatively healthy life until the last six months, when the disease progressed and the patient succumbed to brain metastasis. This case indicates that post-operative chemotherapy can achieve a good effect and prolong the survival of patients, even in those with multiple bone metastases.
